# Hematochezia From Splenic Arterial Pseudoaneurysm Ruptured Into Pancreatic Pseudocyst Coexisting With Fistula to the Colon: A Case Report and Literature Review

**DOI:** 10.14740/gr607w

**Published:** 2014-05-02

**Authors:** Jihong Zhao, Xianglei Kong, Dianbo Cao, Lijuan Jiang

**Affiliations:** a Department of Radiology, The Tumor Hospital of Jilin Province, Changchun 130021, Jilin Province, China; b Department of Radiology, The First Hospital of Jilin University, Changchun 130021, Jilin Province, China

**Keywords:** Chronic pancreatitis, Pancreatic pseudocyst, Splenic pseudoaneurysm, Colonic fistula, Hematochezia, Multislice CT

## Abstract

Activated pancreatic enzymes due to pancreatitis track along anatomic fascial planes and result in digestion of the surrounding tissues and pseudocyst formation. Pancreatic pseudocysts can cause variable complications in some cases. Abdominal contrast-enhanced CT scan can provide a valuable method to identify pancreatic pseudocyst and its related complications, especially in evaluating the adjacent vascular involvement. Splenic arterial pseudoaneurysm ruptured into pancreatic pseudocyst together with fistulous communication with the colon is a very rare condition. So, here we report such an additional case with abruptly acute lower gastrointestinal bleeding on his admission, who was finally diagnosed to be splenic arterial pseudoaneurysm ruptured into pancreatic pseudocyst coexisting with fistula to the colon by contrast-enhanced CT scan and treated successfully by urgent surgery.

## Introduction

Pancreatic pseudocysts are organized non-epithelized fluid collections in the context of acute, chronic pancreatitis or pancreas injury, and have virtually been described in any organ of the body depending upon where activated pancreatic enzymes are released and what path the enzymatic digestion takes. Pancreatic pseudocyst develops in 5% to 15% of cases of acute pancreatitis and in 10% to 20% of cases of chronic pancreatitis [1]. The pseudocyst can cause a variety of complications such as infection, obstruction, bleeding or development of fistula, but of sometimes catastrophic consequences. Erosion of the pancreatic inflammatory process or a related pseudocyst into a peripancreatic vessel may result in pseudoaneurysm formation [2]. Gastrointestinal bleeding may occur if the hemorrhagic pseudocyst ruptures into the gastrointestinal tract and pancreatic duct [3, 4]. Various complications related to pancreatic pseudocysts can be easily observed by multiple-detector contrast-enhanced CT scan [5]. Splenic arterial pseudoaneurysm ruptured into pseudocyst coexisting with fistula to the colon is rarely encountered among those complications of pancreatitis, and this disease entity posed dramatic challenge in the timely diagnosis and optional treatment. Therefore, we report an additional case of pancreatic pseudocyst with such an unusual complication.

## Case Report

**Figure 1:**
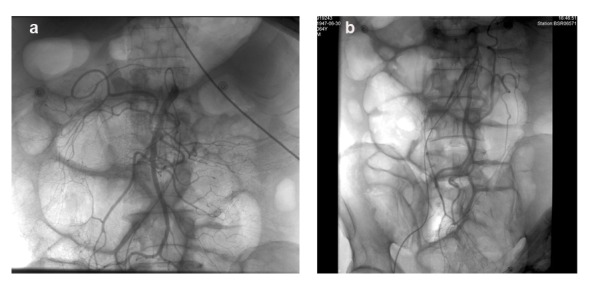
DSA of superior mesenteric artery and inferior mesenteric artery did not reveal any vascular lesions.

**Figure 2:**
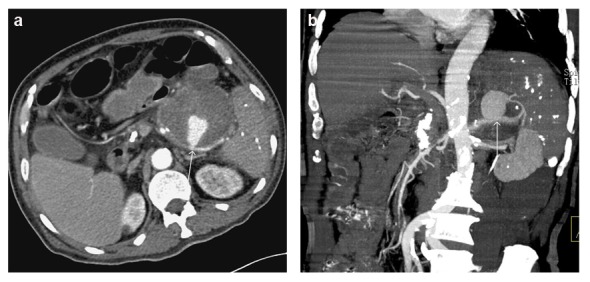
Axial and coronary reconstructed CT images showed splenic arterial pseudoaneurysm and giant pancreatic pseudocyst in the tail of the pancreas.

**Figure 3:**
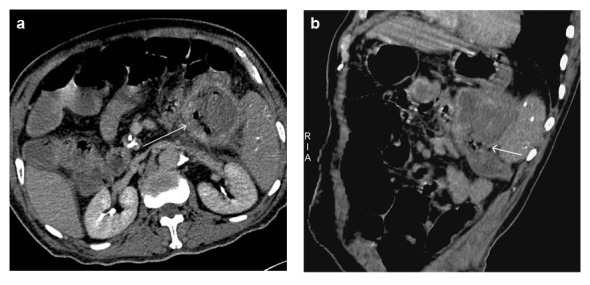
Axial and oblique reconstructed CT images showed ill-demarcated margin with splenic flexure of the colon and the presence of air bubbles within pseudocyst, suggesting the communication between pancreatic pseudocyst and colon.

A 64-year-old male who presented with constipation and diarrhea for 2 days was admitted to our hospital in order to investigate sudden fresh blood in stool around 1,000 mL prior to 1 h. At the same time, he was associated with dizziness and fatigue. The patient was diagnosed as acute pancreatitis and was treated conservatively with good outcome 3 years previously. He was a chronic alcohol abuser, consuming about 200 mL of alcohol daily for the preceding 30 years. On physical examination, his heart rate was 90 BPM and blood pressure was 100/70 mm Hg. He appeared pale and there was no abdominal mass found on palpation. Blood routine examination showed decrease of the hemoglobin values down to 8.4 g/L, a clue of the presence of active bleeding. Intravenous fluid administration and whole blood transfusion of 400 mL were administered. During 12 h of hospital stay and bowel preparation for colonoscopy, the patient had experienced six episodes of hematochezia approximating 1,700 mL. Six units of packed red cell transfusion and intravenous fluids were given following these events. Emergent colonoscopy showed a lot of blood clot at the location of colonic splenic flexure 70 cm distal to anus and the colonoscopy was difficult to pass it, but there was no mass or ulcerative lesions detected. Subsequent emergent DSA of superior mesenteric artery and inferior mesenteric artery did not reveal any abnormalities (Fig. 1). Angiography of celiac trunk was neglected because of suspicious bleeding source from lower gastrointestinal tract. Then abdominal contrast-enhanced CT scan was required for searching the source of gastrointestinal bleeding. Axial CT images and its reconstructed images after contrast injection revealed pancreatic pseudocyst and splenic arterial pseudoaneurysm within it (Fig. 2), an uncommon complication of chronic pancreatitis. Simultaneously, partially ill-demarcated margin with colon and presence of scattered air bubbles within pseudocyst were also seen, a direct evidence suggesting communication between pancreatic pseudocyst and splenic flexure of colon (Fig. 3). So, exploratory laparotomy was necessary to be performed immediately.

**Figure 4:**
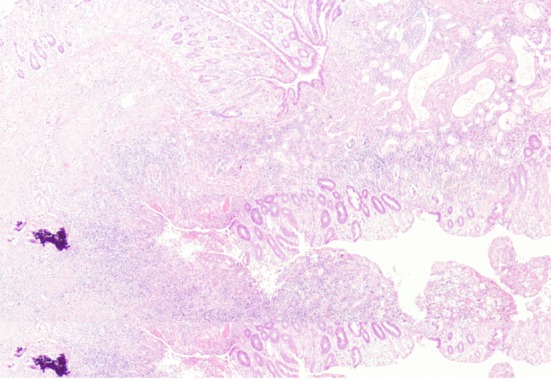
Microscopic findings of resected specimen showed the formation of fistula associated with colonic inflammation reaction.

Intraoperative findings showed cystic and solid mass at the tail of pancreas with irregular shape measuring 10 × 8 × 7 cm. The mass had partially well-circumscribed margin and the rest was adhering to splenic hilum and adjacent segment of transverse colon. The cyst was dissected and was found to communicate with colon. Meantime, there was also opening of splenic artery rupture about 0.2 cm and distal splenic arterial pseudoaneurysm. So, splenectomy, pancreatic tail resection and splenic flexure resection were performed together. Histopathological examination showed pancreatic pseudocyst with chronic inflammation, multiple colonic ulcers and a perforation between pseudocyst and colon (Fig. 4). Post-operative course was uneventful and the patient was discharged from hospital on the postoperative 2 weeks. The patient was free of disease on the 2 years follow-up.

## Discussion

Pancreatic pseudocysts are the most common complication of acute and chronic pancreatitis, mainly because activated pancreatic enzymes in those settings track along anatomic fascial planes causing digestion of the surrouding tissues and leading to distant pseudocyst formation. Pancreatic pseudocysts develop in 5% to 15% of cases of acute pancreatitis and in 10% to 20% of cases of chronic pancreatitis [1]. The pseudocyst can cause a variety of complications such as infection, obstruction, bleeding or development of fistula, but of sometimes catastrophic consequences [6, 7]. Rarely, erosion of the pancreatic inflammatory process or a related pseudocyst into an adjacent vessel results in pseudoaneurysm formation, the relative common for those vascular accidents. The incidence of pseudoaneurysm formation in patients with pancreatitis has been estimated at up to 10% [8], and most pseudoaneurysms develop in association with, and in close proximity to pseudocysts. Although any peripancreatic vessel can be involved, the most common site is still splenic artery [9]. Severe bleeding may occur if the hemorrhagic pseudocyst ruptures into the gastrointestinal tract, peritoneal cavity, retroperitoneum or pancreatic duct. In above these circumstances, the gastrointestinal tract is the most frequently affected locations and rupture of pseudoaneurysm into those sites may take place directly or indirectly after intracystic rupture. In our case, lower gastrointestinal bleeding mainly originated from pseudocyst fistula to colon after intracystic ruputure of pseudoaneurysm.

Gastrointestinal bleeding from a pancreatic pseudocyst relevant to splenic pseudoaneurysm is a rare condition posing diagnostical and therapeutical challenges [10]. For massive gastrointestinal tract bleeding of unknown etiology (defined as persistent or recurrent bleeding in the face of negative upper endoscopy and colonoscopy), especially in patients with a previous history of pancreatitis or pseudocyst, pancreatic diseases should be kept in mind as a possible cause. Intravenous contrast CT can produce excellent opacification of the arteries and veins around the pancreas, and is a valuable method to identify vascular complications. Acute and chronic pancreatitis can be diagnosed and the presence of blood within the pancreatic collection or pseudocyst may also be easily noticed [5, 11]. Occasionally, some signs of pancreatic pseudocyst communicating with gastrointestinal tract, such as multiple air bubbles within pseudocyst and ill-defined margin with regional gastrointestinal tract, can be noticed concomitantly. So, multiphase enhanced CT scan should be considered as priority procedure for those patients with suspected pancreatic disease. It is regretful to miss accurate diagnosis when DSA is firstly performed in our case. For gastrointestinal tract bleeding of unknown etiology, multiphase CT scan should be recommended because endoscopy can miss up to 20% of lesions in the upper gastrointestinal tract or colon that can cause gastrointestinal bleeding [12-14]. Whether further DSA is required or not should depend on the outcomes of contrast-enhanced CT, and angiography of celiac trunk, superior mesenteric artery and inferior mesenteric artery must all be included.

The occurrence of splenic arterial pseudoaneurysm in those patients with pancreatic pseudocyst is the uncommon cause for abrupt acute gastrointestinal bleeding. Concerning the treatment of splenic pseudoaneurysm, it should be carried out according to the patient’s condition, presentation of the disease and availability of treatment modalities. Many studies have shown effective and persisting control of bleeding pseudoaneurysm by transcather arterial embolization, and this procedure has been used as the first treatment in hemodynamically stable patients [10, 15]. But for complicated conditions similar to our patient, only selective arterial embolization cannot achieve permanent outcome because the communication between pseudocyst and colonic splenic flexure will yield to high incidence of fulminating sepsis or recurrent massive hemorrhage. The fistula of a pancreatic pseudocyst to the gastrointestinal tract must be considered in different ways. If it occurs without hemorrhage into the stomach or small bowel, there may be complete drainage and the problem may solve itself. However, the fistula of a pancreatic pseudocyst to colon, especially in combination with massive bleeding from pseudoaneurysm, is the most dangerous condition. Although literatures have shown good response of embolization, surgical treatment still is considered as a mainstay for the treatment of this disease entity [16, 17]. Surgical options include splenectomy with or without distal pancreatectomy, ligation of splenic artery with resection of the pseudoaneurysm and transcystic ligation of the bleeding vessel with internal and external drainage of the pseudocyst. Embolization was not candidate in our case, so splenectomy, distal pancreatic resection and colonic splenic flexure removal were together implemented with a good prognosis.

In summary, this case highlights that any patient with the history of pancreatitis presents with obscure gastrointestinal bleeding, complications of pancreatic diseases should be kept in mind as a possible cause. A careful evaluation on a thin-sliced contrast-enhanced CT images and its reconstructed images can provide strongly diagnostic clues. For splenic arterial pseudoaneurysm ruptured into pancreatic pseudocyst concomitant with a fistula to the colon, radical surgery is the optional strategy for controlling gastrointestinal bleeding.
